# Stigma and Behavior Change Techniques in Substance Use Recovery: Qualitative Study of Social Media Narratives

**DOI:** 10.2196/57468

**Published:** 2025-03-26

**Authors:** Annie T Chen, Lexie C Wang, Shana Johnny, Sharon H Wong, Rahul K Chaliparambil, Mike Conway, Joseph E Glass

**Affiliations:** 1 Department of Biomedical Informatics and Medical Education University of Washington School of Medicine Seattle, WA United States; 2 Department of Linguistics University of Washington Seattle, WA United States; 3 School of Nursing University of Washington Seattle, WA United States; 4 Northwestern University Feinberg School of Medicine Chicago, IL United States; 5 School of Computing and Information Systems University of Melbourne Melbourne Australia; 6 Kaiser Permanente Washington Health Research Institute Seattle, WA United States

**Keywords:** stigma, substance use, transtheoretical model, behavior change techniques, social media

## Abstract

**Background:**

Existing literature shows that persons with substance use disorder (SUD) experience different stages of readiness to reduce or abstain from substance use, and tailoring intervention change strategies to these stages may facilitate recovery. Moreover, stigma may serve as a barrier to recovery by preventing persons with SUDs from seeking treatment. In recent years, the behavior change technique (BCT) taxonomy has increasingly become useful for identifying potential efficacious intervention components; however, prior literature has not addressed the extent to which these techniques may naturally be used to recover from substance use, and knowledge of this may be useful in the design of future interventions.

**Objective:**

We take a three-step approach to identifying strategies to facilitate substance use recovery: (1) characterizing the extent to which stages of change are expressed in social media data, (2) identifying BCTs used by persons at different stages of change, and (3) exploring the role that stigma plays in recovery journeys.

**Methods:**

We collected discussion posts from Reddit, a popular social networking site, and identified subreddits or discussion forums about 3 substances (alcohol, cannabis, and opioids). We then performed qualitative data analysis using a hybrid inductive-deductive method to identify the stages of change in social media authors’ recovery journeys, the techniques that social media content authors used as they sought to quit substance use, and the role that stigma played in social media authors’ recovery journeys.

**Results:**

We examined 748 posts pertaining to 3 substances: alcohol (n=316, 42.2%), cannabis (n=335, 44.8%), and opioids (n=135, 18%). Social media content representing the different stages of change was observed, with the majority (472/748, 63.1%) of narratives representing the *action* stage. In total, 11 categories of BCTs were identified. There were similarities in BCT use across *precontemplation*, *contemplation*, and *preparation* stages, with social support seeking and awareness of natural consequences being the most common. As people sought to quit or reduce their use of substances (*action* stage), we observed a variety of BCTs, such as the repetition and substitution of healthful behaviors and monitoring and receiving feedback on their own behavior. In the *maintenance* stage, reports of diverse BCTs continue to be frequent, but offers of social support also become more common than in previous stages. Stigma was present throughout all stages. We present 5 major themes pertaining to the manifestation of stigma.

**Conclusions:**

Patterns of BCT use and stigmatizing experiences are frequently discussed in social media, which can be leveraged to better understand the natural course of recovery from SUD and how interventions might facilitate recovery from substance use. It may be important to incorporate stigma reduction across all stages of the recovery journey.

## Introduction

### Background

As of 2022, among individuals aged ≥12 years in the United States, 17.3%, or 48.7 million people, met the diagnostic criteria for substance use disorder (SUD) in the last year [1]. Considering individuals needing treatment for SUD in the past year, it is estimated that only approximately one-fourth received treatment [2].

SUD is a highly stigmatized condition [3], and there is considerable evidence that individuals experiencing SUD are faced with stigmatizing experiences in their communities, at work, and in health care contexts [4]. Because stigma substantially worsens outcomes for individuals with SUD [5], mitigating stigma’s effects is important in improving outcomes for individuals experiencing SUD.

A number of models have been developed to understand health behavior change [6]. In the context of SUD and addiction recovery, the most widely used theory is the transtheoretical model (TTM) [7]. The TTM was originally developed in the 1980s in the context of designing smoking cessation interventions and remains a dominant model for conceptualizing recovery phases [8]. The theory posits that individuals experiencing SUD can vary in their readiness to change a given health-related behavior and that determining a person’s “change readiness” can facilitate the choice of an appropriate intervention. As such, successful behavior change is a *process* that takes place over time, rather than a single decision at a specific point in time. According to the TTM, an individual experiencing SUD can be located in 1 of 6 stages: *precontemplation* (ie, not intending to make a behavior change within the next 6 months), *contemplation* (ie, intending to make a change within the next 6 months), *preparation* (ie, intending to take action within the next 30 days), *action* (ie, making active, sustained attempts at behavior change for less than 6 months), *maintenance* (ie, keeping up a sustained behavior change for more than 6 months), and *termination* (ie, completed behavior change, with no desire to return to the previous behavior). In addition to the stages of change, the TTM argues that particular processes of change are effective for individuals at different stages, thus serving as recommendations for intervention design.

However, in recent years, there has been increased awareness of the need to better understand behavioral interventions, particularly due to the rapid evolution of technologies that are often used to deliver interventions [9]. The behavior change technique (BCT) taxonomy [10] is a resource that has been developed to serve as a method for specifying interventions and the components that account for their effectiveness. The taxonomy includes a standardized set of 93 BCTs proposed to be “active ingredients” of interventions (eg, personalized feedback about health behaviors, self-monitoring of symptoms and behaviors, and reinforcement of positive change).

Social media and other forms of consumer-generated textual data are a widely used resource for understanding health behaviors and serve as a useful complement to more established data collection methods (eg, surveys and qualitative interviews) [11,12]. A key advantage of social media as a source of data is that they provide spontaneous, naturalistic, unmediated, and first-person accounts of individual user’s feelings and attitudes, which can provide insights that more hypothesis-driven data collection strategies may not necessarily capture [13].

Reddit is a large, predominantly text-based social media site organized into subreddits (ie, forums centered on a common theme or topic) in which users pseudonymously create posts and respond to other users’ posts. Compared to short text microblog services such as Twitter (rebranded to X), video-based services (eg, YouTube and TikTok), and image-based services (eg, Pinterest and Instagram), Reddit encourages a more verbose, discursive style of interaction, often including rich, detailed narratives that share users' experiences, foregrounding the voices and experiences of individuals living with SUD. As such, Reddit has been extensively used as a resource for investigating substance use in the context of smoking cessation and vaping [14-16], cannabis use [16-18], alcohol use [19,20], and opioid use [21,22].

### Objectives

With this paper, we aimed to build on existing work that uses Reddit data to explore social media users’ experiences of using (and ceasing to use) alcohol, cannabis, and opioids. We selected these 3 substances because public perception differs for different substances [23]. The stigma associated with the use of a substance can vary due to factors such as perceived dangerousness, social acceptance, and legality [24], and these substances may complement one another in terms of the insights offered with respect to people’s use experiences and recovery strategies. Moreover, alcohol and cannabis are among the most widely used substances in the United States [25], and there was widespread concern about the opioid crisis during the study period [26,27]. We used qualitative data analysis methods to characterize the behavior change strategies that may be used at different stages of the TTM and explored a challenge that Reddit authors report experiencing throughout the stages—stigma relating to substance use. Our investigation was framed around 3 research questions (RQs):

- RQ1: What are the manifestations of the stages of the TTM among Reddit authors?
- RQ2: Which BCTs emerge organically at different stages of the TTM?
- RQ3: How do participants experience stigma, and how do these experiences of stigma affect recovery?

## Methods

### Data Collection and Preparation

**Figure 1:**
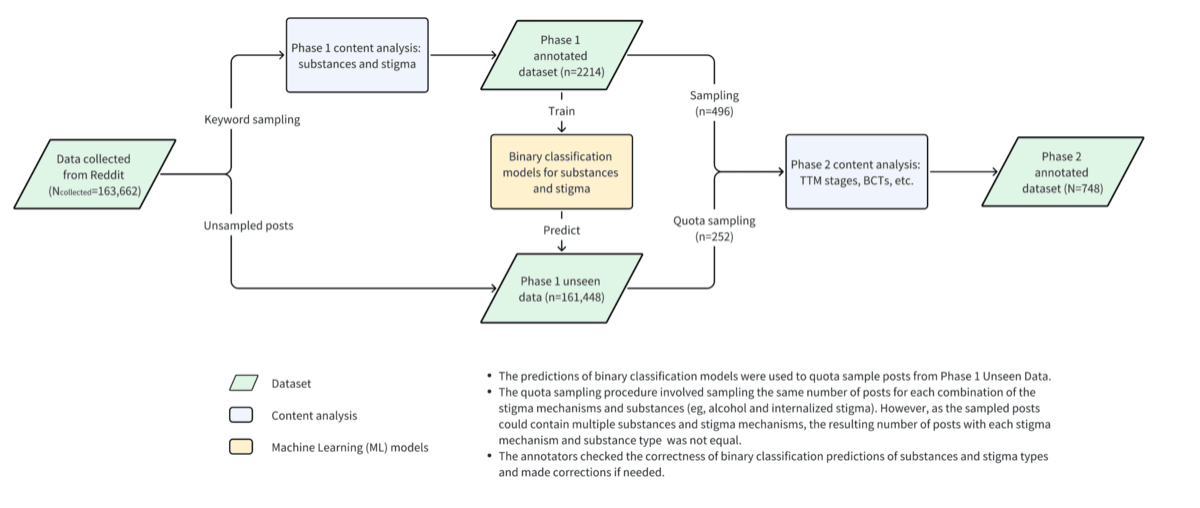
Data preparation flowchart illustrating the process of identifying subreddit posts containing narratives of stigma and recovery related to alcohol, opioid, and cannabis use. BCT: behavior change technique; TTM: transtheoretical model.

The data collection and preparation process included multiple phases (Figure 1). In phase 1, as described in a previous study [28], we collected data from different subreddits relating to alcohol, cannabis, or opioid use (N=163,662 posts) using the pushshift.io application programming interface [29]. In the selection of subreddits, we sought to be inclusive of orientation toward use, including those more oriented toward recovery (eg, r/stopdrinking, r/leaves, and r/OpiatesRecovery) and those adopted various other positions regarding consumption and use (eg, r/alcohol, r/cripplingalcoholism, r/Marijuana, r/Petioles, r/trees, r/cannabis, r/opiates). The posts were authored between January 1, 2013, and December 31, 2019. Data collected from subreddits only included “initiating” posts, as previous work has reported that these posts tend to contain richer narratives than replies to the initiating post [30].

We then performed content analysis on a subset of the corpus (2214/163,662, 1.35%) sampled using keywords. Given the salience of stigma in substance use recovery, posts were eligible if they contained keywords suggesting that stigma might be present, including adjectives that are highly indicative of stigma (eg, “shame,” “untrustworthy,” and “disappoint”), pejorative labels (eg, “crackhead,” “junkie,” and “alcoholic”), and references to persons who might be involved in stigma-related experiences (eg, partners, parents, and coworkers). In phase 1 content analysis, we annotated the substances used and stigma mechanisms present (ie, internalized, anticipated, and enacted stigma). These procedures are described in detail in a prior study [31].

Building on this foundation, we developed a set of binary classification models using the phase 1 annotated dataset as training data. The models use machine learning (ML) algorithms to differentiate between the presence versus absence of substance use and stigma mechanisms. Architecturally, the models combine an autoencoder RoBERTa framework [32] and a multilayer perceptron that takes features that may be informative of stigma as input. The features include Term Frequency-Inverse Document Frequency weighted n-grams (ie, the n-grams are weighted based on their importance in a given post, relative to the corpus), affective and psychological lexical features, and additional handcrafted features. A detailed description of the development and evaluation of the classifiers is available in a prior study [33]. These models were then used to generate ML predictions of substances and stigma on the unseen data (161,448/163,662, 98.65%).

In phase 2, we performed qualitative data analysis to delve deeper into Reddit authors’ experiences of stigma and the behaviors they engaged in while seeking to recover from substance use. To enrich the concentration of stigma in our sample, we sampled the first 496 posts in the phase 1 annotated dataset consisting of posts with and without stigma and combined this with another sample of 252 posts selected from the unseen data. Post selection was performed using the previously mentioned ML predictions of stigma and substances, with the goal of sampling equal proportions of the 3 stigma mechanisms and the 3 substances. Using this procedure, we were able to ensure that the resulting dataset for this study (N=748) contained sufficient posts relating to the 3 substances and a varied distribution of posts with and without stigma for subsequent qualitative analyses.

### Data Analysis

We performed a qualitative analysis of the posts using a hybrid inductive-deductive method, which seeks to leverage the strengths of each respective approach [34]. The deductive or “top-down” part of our analysis was based on 2 theoretical frameworks and a taxonomy: the stigma framework [31,35], the stages of change from the TTM [7], and BCTs as defined in the behavior change taxonomy [10]. We also included the substances of interest.

As noted by Proudfoot [34], using a hybrid inductive-deductive approach to analysis “helps to ensure that the voices of the participants are valued, while simultaneously allowing for more theory-led analysis.” Given the sensitive nature of our research focus and the richness of our dataset, adopting an inductive component was particularly important for ensuring that these voices are heard. Thus, our codebook development included an inductive or “bottom-up” component in which we adapted the definitions to our data to account for the specifics of the health behavior context and data type. For example, though the stage of *action* has been defined as having modified their lifestyles within the past 6 months [7], often there is no explicit declaration of time in the social media data. Thus, to differentiate *action* from the next stage in the TTM, *maintenance*, our coding guide specifies that situations where social media authors appeared to be actively seeking to quit substance use are indicative of the *action* stage, whereas situations where users maintain routinized strategies are characteristic of the *maintenance* stage.

In developing codes for BCTs, we focused on the high-level groupings of the taxonomy [10], such as “antecedents,” which include more specific techniques, such as “restructuring the physical environment” and “restructuring the social environment.” Though the BCT taxonomy does not differentiate between social support seeking and offering, we chose to do so due to the diverse ways that social support has been studied in the literature [36] and the potential importance of differentiating support sought and received in terms of health outcomes [37]. The taxonomy includes 16 high-level groups; we only included a subset of these because the others did not appear salient in our data.

We also coded the substances of interest (eg, alcohol, cannabis, and opioids) and the stigma mechanisms encountered, identified either through qualitative coding or using the models and checked by the annotators, as described previously. The stigma mechanisms—enacted, anticipated, and internalized stigma—were adapted from the stigma framework [31,35]. Enacted stigma describes experiences of stereotyping, prejudice, and discrimination due to a stigmatized attribute; anticipated stigma is the expectation that one might experience stereotyping, prejudice, and discrimination in the future due to a stigmatized attribute; and internalized stigma is the application of negative feelings and beliefs about a stigmatized group to oneself (Table 1) [38]. For the stigma mechanisms, we also identified the actors involved (eg, family, friends, partners, coworkers, others, society, and self).

Our annotation process involved 2 primary coders (SJ and RKC), who independently coded the discussion posts; an adjudicator (SHW), who reviewed the codes and facilitated reconciliation; and 1 additional investigator (ATC), who provided additional feedback as needed. All disagreements were reconciled by majority or consensus. Interrater reliability for the substance and stigma codes are reported in a prior study [33]. For the stages of the TTM and BCTs, we assessed interrater reliability using Cohen κ [39]; overall interrater reliability was 0.71 between the 2 coders. Dedoose qualitative data analysis software was used to perform the coding [40]. For each research question, we report our findings using descriptive statistics as well as narrative descriptions of each theme.

**Table 1:** The stigma framework: definitions and examples.

	Definition	Example
Enacted stigma	Experiences of stereotyping, prejudice, and discrimination from others due to a stigmatized attribute.	“My husband called me an addict and said I’d never become clean.”
Anticipated stigma	The expectation that one might experience stereotyping, prejudice, and discrimination from others due to a stigmatized attribute.	“My co-workers are giving me looks now at work after I went in drunk last week. I don’t know how I’m going to face them day after day.”
Internalized stigma	Individuals internalize negative stereotypes about the stigmatized group that they belong to and apply these to themselves, affecting their sense of self-worth.	“I’m a stoner. I am an awful person...”

### Ethical Considerations

Social media can offer naturalistic, first-person accounts of individual user’s feelings and attitudes, allowing for understandings that hypothesis-driven data collection strategies may not necessarily capture [13]. Moreover, prior literature has reported that social media authors often believe social media content is public domain and can be freely used in research without consent as long as individual information is not reported [41].

However, particular care must be taken to protect the identities and ensure the anonymity of content authors [42]. In this study, we analyzed the collected data without attempting to contact or connect social media authors to their posts; then, we used synthetic or paraphrased quotations based on the original social media content to illustrate the themes we present. As there are situations in which search engines might be used to retrieve social media posts [42], we constructed synthetic quotations to protect the identities of content authors.

In the development of these synthetic quotations, we seek to faithfully represent the voices of social media authors while protecting their identities by selecting original quotations that are representative of the data [43], faithful representation of concepts in the synthesized quotations, and using language that resembles language in the source medium to maximize authenticity. As we did not seek to connect social media authors to their posts, we also did not compensate them. This study has received ethics approval from both the University of Washington Human Subjects Division (STUDY00015737) and the University of Melbourne Human Research Ethics Committee (2023-25512-48127).

### Reflexivity Statement

Our analysis incorporates many recommended practices for establishing rigor and trustworthiness in qualitative research [44]. We ensured thick, rich descriptions using a sampling strategy that leveraged the large volume of available data while maintaining our specific research focus, and through a method of synthetic quotation creation. We performed *triangulation* during the data analysis and reporting process through the use of multiple coders of different backgrounds, including those with clinical experience (SJ and RKC) and informatics experience (ATC and SHW) and an investigator with significant experience in qualitative research, analysis of social media data, and the lived experiences of persons with chronic conditions (ATC). An important part of the annotation and synthetic quotation creation process involved discussion among the research team to ensure consistent coding over time, faithfulness to the data, and contextualization of the data considering the complementary backgrounds of the researchers. The discussion had several purposes, including consideration of different perspectives and mitigating the potential bias of any 1 researcher. Last but not least, our procedures facilitate *auditability* through detailed notes on our data preparation process and annotation process.

## Results

### Sample

Our sample comprised 748 posts pertaining to 3 substances: alcohol (n=316, 42.2%), cannabis (n=335, 44.8%), and opioids (n=135, 18%). Each post relates to at least 1 substance. On average, posts were 348 (SD 315) words long. Internalized stigma (369/748, 49.3%) was the most commonly expressed across all stages, but anticipated (131/748, 17.5%) and enacted stigma (111/748, 14.8%) were also present.

### RQ1: How and to What Extent Are the Posts Relating to Each Stage Represented in the Sample?

The posts reflected different stages of change (Table 2). A subset of the posts was in the *precontemplation* stage, when social media authors do not express an intent to quit. These could include posts in which people are simply talking about experiences that they have had relating to substance use, which could include legal consequences, trouble at work or home, and more, but do not demonstrate an intention to quit. Often in the *precontemplation* stage, internalized stigma manifested as hopelessness or existential questions:

I feel horrible about my life every day. I feel bad about not taking care of my kids but I just keep picking up the bottle.... My life is hopeless.

Am I depressed because I am a drunk or a drunk because I am depressed?

In the *contemplation* stage, social media authors begin thinking about quitting but may identify barriers to doing so:

I’m thinking about quitting again, but I can’t get time off of work to go into a detox program...

They may also reach out to the Reddit community to ask their opinion about whether to quit, how to do so, and other related questions.

In the *preparation* stage, social media authors report taking different steps to quit, including deleting the phone numbers of dealers, planning to enroll in rehabilitation programs, telling friends and family about their use to seek support in the quitting process, and posting on Reddit to keep themselves “accountable.” Many also mention that it is time to stop. At times, there is an event that may trigger the *preparation* stage, such as such as running out of the substance being used or being discovered by parents. Throughout the stages, feelings of depression, despair, and hopelessness are often expressed.

In the data that we annotated, the most commonly represented stage was *action*. In these posts, Reddit authors often shared their experiences with their recovery journeys. These posts often included rich detail concerning challenges that they faced:

I have been thinking about attending meetings but am also worried that the experiences of those worse off will cause me to slip.

The holidays are the worst. I keep thinking that I’ll just have a drink but I know that won’t happen.

In the *maintenance* stage, social media authors often share how long they have been in recovery and seek to encourage others by sharing their experiences:

This morning, I played with my son. I went to the park with him and enjoyed the moment with a clarity that I would have thought impossible a year ago. I’m so grateful that I’m now here. If you are struggling, take it one day at a time. It will get better.

However, not all the narratives in the *maintenance* stage express positive sentiment. Some express continued cravings, feeling like they might relapse, and problems experienced while sober. While some say that they do not feel tempted at all, others say that they do not feel that they will relapse but sometimes reminisce about the effect of their substance of choice:

While I don’t think I’m in any danger of relapsing, sometimes I feel the call of sweet mary jane and look back fondly.

According to the TTM, people often slip back to prior stages. In the social media content, people often reported situations in which they had been sober for some time and then relapsed or are on the point of relapse:

Five months sober. Last month I decided I could have a drink while I was out with my coworkers, and it’s been downhill from there. I have to stop!

Feeling like I might relapse. I have been sober for a year and two months, but my life is as messed up now as before. I don’t feel any better, and now I have no one to talk to. I don’t want to start over again and feel the high heart rate, paranoia, guilt, and lack of motivation all over again, but the desire to smoke is overwhelming.

As presented in Table 2, not all the data were representative of a stage of change. This could happen for various reasons. For example, a person might write a post providing advice but not clearly indicate that they were in recovery or provide commentary on relevant material, such as policy, a movie, or book on how to quit.

**Table 2:** Distribution of the stages of change in the dataset (N=748).

Stage	Posts, n (%)
Precontemplation	95 (12.7)
Contemplation	68 (9.1)
Preparation	43 (5.7)
Action	472 (63.1)
Maintenance	45 (6)
Unknown	25 (3.3)

### RQ2: What BCTs Do People Naturally Use as They Seek to Recover From Substance Use in the Different Stages?

**Figure 2:**
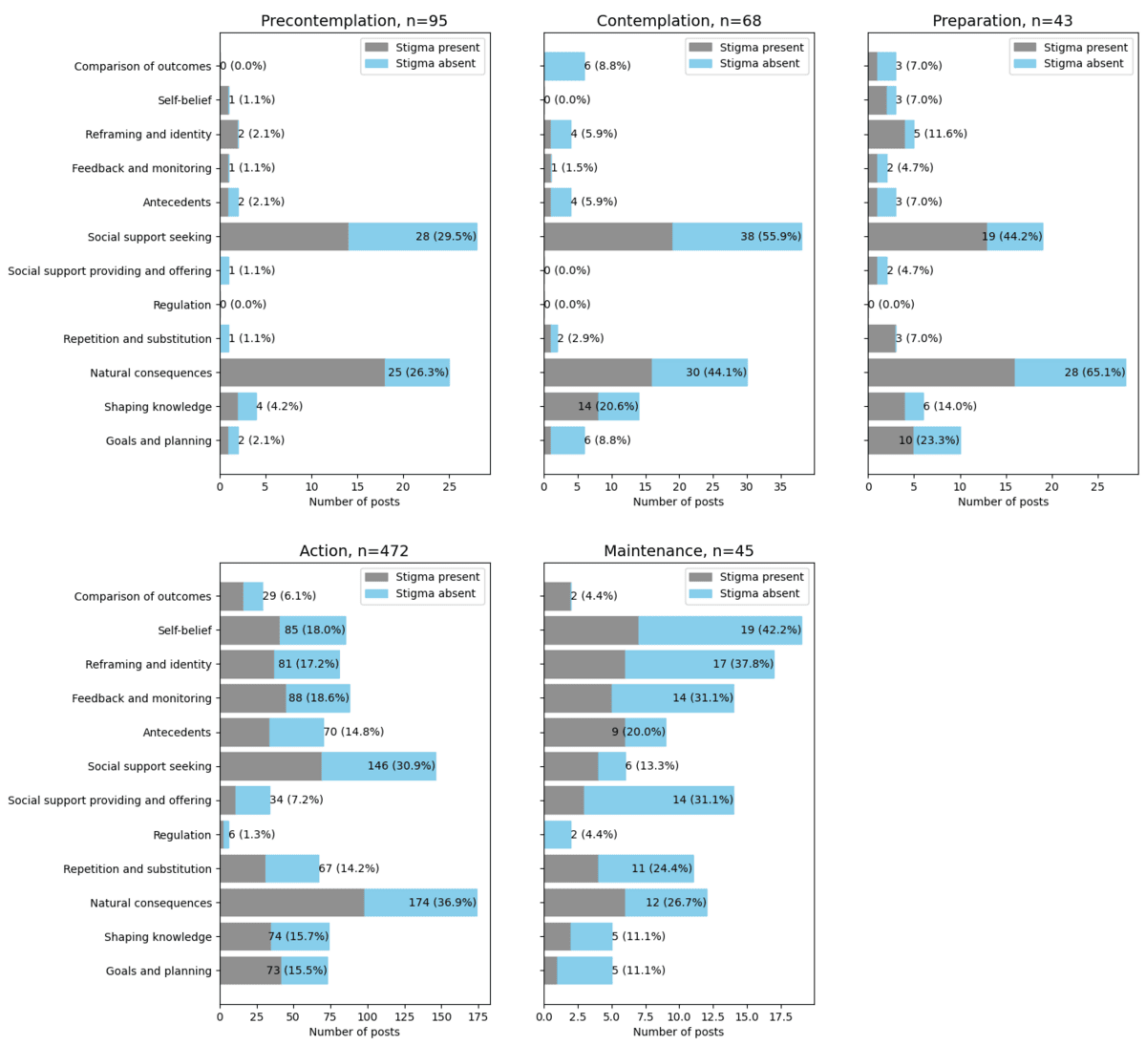
The use of behavior change techniques (BCTs) and the presence of stigma mechanisms in individual stages of change.

In Textbox 1, we present the BCTs observed, along with their definitions and examples. In Figure 2, we depict the prevalence of BCTs observed in the TTM stages, with the length of each bar reflecting the number of posts in which a given BCT was observed. The frequency of BCTs varied for the different stages of change. There were similarities in the *precontemplation*, *contemplation*, and *preparation* stages, with social support seeking and awareness of natural consequences being the most common:

Reddit, help! I have lost everything because of my habit... dropped out of college and am living at home with my parents. I feel like a failure. I need help but I don’t know how to start.

Social support seeking and natural consequences

Posters reported that they sought social support online, in online support groups and other digital and web-based formats such as subreddits, as well as from family, friends, and meetings such as Alcoholics Anonymous.

In the *contemplation* stage, compared to the *precontemplation* stage, we observed a greater proportion of posts including some element of *shaping knowledge* or the acquisition of knowledge and information related to the antecedents of the behavior and ways to change it. At this time, social media authors expressed increased awareness of reasons or triggers for use, including loneliness, boredom, negative thoughts, and daily routines or life situations that make post authors more prone to use:

I want to quit and am looking for any advice you all have. I’ve been smoking for years and recently I’ve noticed all these changes in me that make me want to quit. First, I am anxious all of the time now and I can’t sleep. I have no money and weed is draining my finances. I am also using weed as a coping mechanism for stress, which only makes me more stressed.

Shaping knowledge and natural consequences

In the *preparation* stage, we observed a greater proportion of goals and planning, such as seeking out accountability partners and making accountability posts. Post authors also express commitment to quitting or plans to enroll in treatment or detox programs.

In the *action* stage, as people sought to quit their use of substances, we observed a variety of BCTs. For example, by recognizing that merely having access to a substance would facilitate use, social media authors threw away alcohol and equipment that they used to engage in use, such as pipes. They also enlisted the help of others to ensure that they did not have access to substances and, at times, relocated (antecedents):

Gave away all of the wine in the house.

Antecedents

Put what little I had in my Civic and drove as far as I could south, that was 2 months ago and I haven’t used since, aside from a sub or two for withdrawals. I lost everything when I was using—my friends, my job, my savings, my dignity. No more. Applying to jobs in this new town and planning to play pickup this evening at the local gym. It’s time to rebuild.

Antecedents, natural consequences, and goals and planning

They also sought to engage in repetition and substitution of healthful behaviors, such as exercising while monitoring their own progress (feedback and monitoring):

I’ve started working out regularly and it has helped with the anxiety...

Repetition and substitution

Six days down! I hate what weed has done to me. I am always in a fog and can’t remember what happened yesterday. My heart races. But I am slowly feeling better and clearer. I’m learning what withdrawal is like, trying to give space for my feelings, and gradually getting through it.

Feedback and monitoring

It has been a month since I last smoked. At first, I had a hard time sleeping and was tired all the time. But each day gets better. I have a little more energy. I can think more clearly.

Feedback and monitoring

**Behavior change technique (BCT) groupings, definitions, and examples (Adapted from the behavior change taxonomy [10]).**

**Natural consequences**

- Definition: actions related to the identification of consequences of a behavior to be used as a facilitator of behavior change.
- Identified consequences should not be future, anticipated consequences. Only consequences that were experienced in the past or experienced in the present should be coded.
- Examples: becoming aware of health, social, environmental, and emotional consequences.

**Antecedents**

- Definition: actions that are related to reducing or mitigating antecedents (preconditions) that might lead to the undesired behavior.
- Examples: restructuring the physical and social environment (eg, moving to a new place) and avoidance or reducing exposure to behavioral cues, such as others who engage in substance use.

**Shaping knowledge**

- Definition: actions related to the accumulation of knowledge and information related to the behavior and ways to change it.
- Examples: information about antecedents and instructions on how to perform a behavior.

**Repetition and substitution**

- Definition: actions related to substituting the unwanted behavior with another and rehearsing the performance of a desired behavior.
- Examples: behavioral practice or rehearsal, behavioral substitution, and habit reversal.

**Regulation**

- Definition: actions related to emotional regulation and managing mental and cognitive stress.
- Examples: reducing negative emotions.

**Goals and planning**

- Definition: the identification of behaviors to be changed, creation of goals, and development of plans to facilitate change.
- Examples: reviewing goals and finding accountability partners.

**Social support**

- Definition: actions related to seeking, receiving, or providing social support to assist with behavior change. In this study, we used a broader conceptualization of social support, encompassing both emotional and informational support.
- Examples: general (unspecified), seeking, received, and providing and offering.

**Comparison of outcomes**

- Definition: the identification of potential or realized outcomes as a way to encourage or motivate behavior change.
- Examples: listing pros and cons and persuasive argument.

**Identity**

- Definition: shifting one’s perspective on their past or current identity or actions to encourage behavior change.
- Examples: framing and reframing.

**Self-belief**

- Definition: increasing one’s confidence in successfully performing the desired behavior through reinforcing one’s belief in oneself.
- Examples: mental rehearsal of successful performance, focus on past success, and self-talk.

**Feedback and monitoring**

- Definition: monitoring one’s behavior and the effects of one’s behavior to facilitate the behavior change.
- Examples: self-monitoring of behavior and its outcomes.

Social support is an important part of the quitting process. Social media authors can experience challenges in removing antecedents (ie, triggers) from their environment if others are not supportive:

I have been trying to quit drinking, but a close friend insists on going to the bar whenever we hang out. Every time we go I have a hard time stopping myself from ordering a drink. When I bring this up, he says that I am just weak. What can I do?

Shaping knowledge, antecedents, and social support seeking

Social media authors also sought information from others as to how to maintain the new habits that they tried to adopt, while addressing symptoms that they experience in withdrawal:

The cravings are bad, but what is worse is not being able to sleep. Does that happen to you all? How long do the symptoms last?

Social support seeking

We also observed examples of positive self-talk and reframing, in which participants adopted or attempted to adopt new perspectives:

I’ve reached the two week mark. It hasn’t been easy, but I’m proud of myself, and hopeful that I can do it.

Self-belief

I went to a party and for once, I didn’t make a fool of myself! I had a great time, and was able to focus on every person I talked to...

Self-belief

In the *maintenance* stage, reports of diverse BCTs continue to be frequent. Notably, expressions of self-belief, altered perspective (ie, reframing and identity), and providing support and encouragement to others (ie, social support providing and offering) also become common:

Today I live without shame and look forward to the future. To everyone still struggling out there—you can do it!

Social support providing and offering

Many BCTs that became prominent in the *action* stage, such as self-belief, reframing and identity, feedback and monitoring, and repetition and substitution, remained common or were even observed more often in the *maintenance* stage. While this may partially be due to the nature of the venue—being an online community in which people may strive to be positive to encourage others—this may also underscore the importance of continual reaffirmation and efforts at abstinence:

When I think of my past, I still feel the guilt and shame. But I think of them as a good reminder of what I don’t want to go back to. I also tell myself that my past doesn’t have to define me.

Reframing and identity

As we reflect back on the BCTs used, we observed that there was diversity in the strategies used. For many, it was a combination of BCTs and social support:

I told my partner last night about everything. She was incredibly supportive, and we talked about a plan. I am going to restart therapy. I am worried that I’ll start using again, but for the first time in a long time, I feel hopeful.

Goals and planning, social support seeking, and social support received

As the previous excerpt demonstrates, this process can be a gradual one. While this poster states that others are supportive of their efforts, this is not always the case:

I am in rehab and on subs. I am trying hard to stay clean, but I still smoke weed. I am trying to be a good mother to my kids, but I feel so alone. I have no friends left. I had a friend that I kept up with, but it was too hard to stop using while continuing to spend time with them. I don’t know what to do and would love to have some support.

Antecedents and social support seeking

Social media authors may experience challenges due to their life contexts, including feelings of guilt and other pressures due to family obligations. They may also face challenges dealing with exposure cues in their day-to-day lives. In addition, people may struggle with stress, self-doubt, and loneliness, leading them to ask for others’ opinions on how to deal with these hardships.

### RQ 3: How Do Participants Experience Stigma, and How Do These Experiences Affect Recovery?

#### Overview

In this research question, we aimed to examine how the presence of stigma may affect social media authors’ recovery journeys. In Figure 2, we observed that substantive amounts of stigma (ie, in blue) are present in all stages. In this section, we review key themes involving stigma, which were often observed in multiple stages. We also present stage-specific characterizations as appropriate.

#### Theme 1: Experiencing Enacted Stigma From Others

Social media authors reported experiences of enacted stigma involving different persons that they encounter. Of the different actors that we examined, enacted stigma from family and those who were not part of a person’s social circle was most common. Authors mentioned experiences with pharmacists and physicians discounting health issues, attributing health issues to substance use, and being reticent to provide services when they were aware that participants engaged in substance use. In addition, Reddit authors often spoke of how their use took a toll on their relationships with family, friends, and partners, resulting in broken trust and interpersonal conflict:

I got into a huge fight with my parents. They called me a drug addict and made me feel worthless.

Enacted stigma

One scenario that occasionally arose as social media authors sought to quit substance use was resistance from their social circle. For example, there were examples of those who told their family they wanted to quit drinking, and their family would say that they were not an “alcoholic” or “You’re not that far gone.” Social media authors often had to find new support networks, such as Alcoholics Anonymous, as their family and friends were not always supportive of their efforts to quit.

#### Theme 2: Experiencing Anticipated Stigma

Social media authors also experienced anticipated stigma. Many feared disappointing others, particularly family and friends, or feared repercussions at work, and thus hid their substance use. As many keep their substance use a secret, they may reach out to the Reddit community to ask questions that they are not able to ask elsewhere, often using temporary (ie, “throwaway”) accounts:

This is a throwaway account. I have been addicted to oxy for years and am worried I will lose my job if anyone finds out. I am thinking of taking suboxone but am wondering if it would be better to quit cold turkey or would the withdrawals be worse?

Anticipated stigma

Some authors reach out on Reddit asking whether they should disclose their use to others around them. There are also times when they do choose to do so and are surprised to find that others are empathetic and supportive:

I have been feeling so ashamed and guilty, but I finally told my parents about my drinking and they were surprisingly supportive. I am beginning a rehab program tomorrow.

Goals and planning, internalized stigma, and anticipated stigma

Similar to enacted stigma, Reddit authors most often anticipated stigma from family and others outside of their social circle.

#### Theme 3: The Challenges of Internalized Stigma

There were posts that expressed internalized stigma, including internal struggles that people experienced due to regret, guilt, shame, and disappointment:

I have been able to avoid drinking when others are, but my biggest challenge is when I am alone and start to have negative thoughts and sink into this pool of shame and disappointment. Then I think about what a failure I am and how I have let everyone down.

Internalized stigma

Social media authors often shared challenges that they experienced adjunctive to substance use, such as financial difficulties, poor mental health, and increased social isolation. In the *contemplation* stage, social media authors often speak of “hating themselves” and hesitate to take steps forward due to lack of confidence in their own ability to succeed. In contrast, in the *maintenance* stage, social media authors share that they feel less guilt and have more compassion for themselves:

I always felt this deep self-loathing each time I slipped. I didn’t think I would ever make it. But little by little, I am learning to have compassion for myself. Life is still hard, but it gets a little easier one day at a time.

Internalized stigma

The process of recovery was gradual, and there could be setbacks:

I started out the year in constant shame. I was taking a quick sip whenever I had the chance, and then worrying that I was messing up my child. My mom, who is an alcoholic, came to visit, setting off a three-day bender. Now I am back on track. I haven’t had a drop for a week and I haven’t even been tempted. But I have some stressful stuff coming up at work and am worried. Thanks for listening.

Feedback and monitoring, natural consequences, and internalized stigma

As these posts illustrate, there can be triggers due to work and interpersonal interactions, and people may try for some time, intermittently experiencing setbacks, before being able to sustain abstinence.

#### Theme 4: Multiple Forms of Stigma

There were also examples of multiple forms of stigma in the same post. Shame, guilt from letting others down, and broken trust leading to tolls on relationships with others were common challenges:

I have lost family and friends due to my drug use. My relatives hide their wallets whenever I go over. I feel terrible that no one trusts me, and the worst failure ever.

Enacted stigma and internalized stigma

In earlier stages, people often report hiding their substance use from others and experiencing feelings of negative self-worth from doing so:

I hide my stash from my husband. The other day my five-year old accidentally found it and I was mortified. I feel horrible about hiding this but I don’t think I can stop...

Anticipated stigma and internalized stigma

As illustrated by the quotation above, people may feel low self-worth. As a result, they may also avoid others or be excluded by others (enacted stigma), resulting in social isolation.

#### Theme 5: Enacted Stigma Directed Toward Others

Social media authors also demonstrated enacted stigma toward others. One common situation was when they referred to family members using pejorative labels such as “addicts” or “alcoholics,” which occurred throughout the stages. Another example was a lack of empathy for others among some who felt that they were succeeding in their recovery journeys (ie, often occurring in the *action* or *maintenance* stages):

As I stopped smoking, I came to look down upon the behavior of my stoner friends...

Enacted stigma

In moments of introspection, some individuals reflected on their own behavior, seeking input from fellow Redditors while contemplating their motivations. For example, 1 social media author mentioned that they wanted to help others who were struggling but also felt it is impossible, and so instead chose to distance themselves.

## Discussion

### Principal Findings

"In this study, we performed qualitative data analysis to identify the stages of change and BCTs used by social media authors in various stages of readiness to reduce their use of the following substances: alcohol, cannabis, and opioids. There were some similarities in the use of BCTs in the earlier stages of change (ie, *precontemplation*, *contemplation*, and *preparation*). In posts with content that reflected the *contemplation* stage, authors described an awareness of the negative impacts that substance use had on their lives (ie, natural consequences), and as they prepared to quit, they increasingly took concrete steps to do so by sharing their decision with their social network and committing to it (ie, social support seeking, goals, and planning) and seeking help in other ways.

Posts in the *action* stage reflected that individuals leveraged other BCTs, such as modifying their daily routines (ie, repetition and substitution), modifying their physical and social environment (ie, antecedents), self-affirmation (ie, self-belief), and viewing their situation through new lenses (ie, reframing and identity). Substantive use of BCTs are observed, and in some cases more prominently, in the *maintenance* stage. This may partly stem from the nature of online discussions, where many individuals aim to give back to the community by sharing their experiences with successful health behavior change, offering encouragement to those trying to quit.

We also examined the role of stigma in social media authors’ recovery journeys. We observed substantive amounts of stigma across all stages of change. The influence of social interactions was also salient. Social media authors shared situations in which they experienced or anticipated stigma from strangers, acquaintances, and loved ones. When people receive support from others, this can be affirming and facilitate recovery; however, social isolation can also result from isolating oneself or from enacted stigma. Some social media authors reported that the fear of disappointing others prevented them from reaching out for support. In contrast, other authors sought support from both online and offline social networks (eg, family and friends) and reported that this support was instrumental in successful recovery journeys. Previous research has found that perceived stigma is associated with poorer mental health, while perceived social support is linked to better mental health [45].

### Comparison With Prior Work

This study extends the existing literature concerning recovery mechanisms by identifying situations in which commonly used BCTs appeared in social media narratives, thus affording insight into how people may seek to address the challenges of recovery in everyday life. Consistent with existing literature, which has identified controlling one’s environment and social support as commonly used strategies for behavioral change in the context of substance use [46,47], we observed examples of persons seeking the support of others (*social support seeking*) and using *antecedents*. These including discarding equipment necessary for substance use behavior and physically removing themselves from temptations. However, posters also lamented the difficulties of encountering “triggers” or cues in their day-to-day life that provoked relapse. The use of the antecedents BCT is also common in evidence-based substance use treatments [48], and a prior systematic review found that remotely delivered substance use interventions [49] incorporating intervention components related to “avoidance/reducing exposure to cues for behavior,” “pros and cons,” and “self-monitoring of behavior” may be effective. Social media authors also sought to restructure their lives to mitigate exposures and observed the effects of behavioral change.

In this study, we also observed that self-belief and cognitive reframing (changing one’s perspective), were more common in the *action* and *maintenance* stages. As recovery trajectories progress, there is a trend toward individualistic and agentic identity [50]; BCTs such as self-belief and cognitive reframing may facilitate this process.

Our findings also illustrate the importance of temporality and flux in the experience of the person in recovery, who is not static but rather evolving due to their experiences over time. At times, people may struggle with low self-worth due to internalized and anticipated stigma, impeding their path to recovery; at other times, one might also observe enacted stigma from them toward someone else, often when the person is in recovery. There is also a need for more research concerning how family and romantic partners may also enact stigma [51].

### Practice Implications

These findings have implications for the design of stigma reduction interventions to promote substance use recovery. Textbox 2 summarizes the key themes identified, along with implications for subsequent intervention design. For instance, to counter stigma and feelings of isolation, interventions could provide education to improve self-advocacy and emotion regulation. In particular, treatments such as acceptance and commitment therapy could potentially combat internalized stigma by assisting individuals with acceptance and compassion toward the self [52-54].

**Practice implications considering the role of stigma in recovery journeys.**

**Experiencing enacted stigma from others**

- Provide persons with substance use disorder (SUDs) training in navigating challenging situations involving enacted stigma (eg, in self-advocacy with providers and emotion regulation in everyday situations).
- Assist persons with SUDs to develop alternative support networks and other mechanisms to nurture resilience.

**Experiencing anticipated stigma**

- Promote the use of behavior change techniques (BCTs) encouraging persons with SUDs to develop and strengthen support mechanisms.
- Teach strategies for disclosure of topics related to one’s SUD.

**The challenges of internalized stigma**

- Emphasize self-compassion.
- The internal struggles and gradual process were significant obstacles. Intervention components that serve as a toolkit for participants to combat these challenges, such as feedback and monitoring and comparison of outcomes, can perhaps bolster natural mechanisms.

**Multiple forms of stigma**

- Use approaches that address the different stigma mechanisms individually but also emphasize that the process of recovery involves rebuilding on multiple fronts.

**Enacted stigma directed toward others**

- Assist persons with SUDs to consider the situations of others with an open countenance.

In addition, interventions could encourage the development of new support networks or teach strategies to intentionally seek support from others. Doing so could lessen the impact of the stigma while developing a social network that is supportive of one’s recovery. Given that family interactions were a common source of stigma, interventions could teach strategies to encourage positive interactions with family members or facilitate development of additional support networks outside the family as needed. These study findings show that when family members were supportive, participants valued the support of those that they were close to.

Reddit authors also struggled with internalized stigma and challenges involving multiple forms of stigma, in which authors felt bad about their interactions with others. Interventions that assist persons experiencing self-stigma to disclose and seek support from others could be helpful [55].

Given that this study focused on occurrences of stigma expressed by people who were engaged in online forums, it is also worthwhile to consider how these findings have implications for the design of internet-based interventions. Reddit authors often mentioned the improvements that they observed over time. These improvements seemed to increase their confidence and hope that they would continue to improve. There may be an opportunity here to leverage mobile health apps to facilitate self-monitoring and tracking of incremental improvements. Previous research has also recognized the value of using wearables to monitor biologic data, such as blood alcohol concentration sensors [56], and self-monitoring surveys can help participants reflect on associations between self-monitoring domains and adhere to drug use reduction goals [57]. These are potentially fruitful directions for future research.

The findings from this study could also improve the degree of personalization of content, such as delivering stage-matched intervention content. For example, the results suggest that *shaping knowledge* (information about changing a behavior) is more prominent in the *contemplation* stage as opposed to *precontemplation*, and that people use diverse BCTs as they actively seek to quit substance use. Thus, it may be beneficial to present content that encourages thoughts about antecedents or other methods of shaping knowledge to individuals in the *contemplation* stage, while offering suggestions for different BCTs to those in the *preparation* or *action* stages. In the *maintenance* stage, we observed that some individuals faced the potential for relapse, and other perceived no improvement in their lives. Thus, there may be a need for intervention content tailored to particular challenges of maintenance, such as the position of abstinence as opposed to moderation or maintaining recovery in the context of other aspects of life.

Finally, prior literature has observed that those who engage in social media value the information and support from the online communities [28,36,58,59]. In this study, we observed that social media authors experienced different information and support needs in different phases of their recovery journey. During the *action* phase, social media authors were likely to have questions about the quitting experience, including strategies, withdrawal symptoms, and timing of those symptoms. At all phases, people may experience questions about social interactions, such as “protecting their quit” and changes in how they may view and relate to others as they engage in maintenance. Thus, insights from these study findings can inform the provision of appropriate information at different stages of the recovery journey.

### Limitations

This study has various limitations. First, as this study was based on social media data, there is bias as to who is represented due to differences in people’s decisions about which media to use [60]. However, as indicated by the sample posts, social media authors shared their challenges with substance use in diverse life contexts, including home, work, and school contexts. For example, some social media authors experienced concern with the effect that their use might have on their children, while others described their fears and anxieties with respect to work repercussions. Thus, the study findings still have implications for the diverse life contexts in which people may confront and seek to apply strategies for recovery.

In addition, this study used sampling approaches that sought to increase the proportion of posts containing stigma in the sample. As such, the posts in our sample may not represent the natural distribution of stages of change in substance use–related content on Reddit. There was less data available on stages other than *action*, and there is a need for more research concerning information and support needs of persons with SUDs in the earlier phases. Finally, in this study, we used content analysis to identify different stages of change to characterize BCT use in the different stages. The study does not examine longitudinal changes in the same person over time, and this could be an area for future exploration.

### Conclusions

This study used social media as a naturalistic data source to explore how people may use BCTs to recover from substance use, including how this use may vary depending on a person’s readiness to quit or reduce their substance use. We observed that there were differences in the BCTs that were most commonly used in the different stages. Stigma was present in a substantive portion of the narratives at all stages, suggesting that there is a need to incorporate intervention components to assist participants address stigma-related challenges. Consideration of these patterns might be used to personalize and deliver content in future interventions promoting substance use recovery.

## Abbreviations

BCT: behavior change technique
ML: machine learning
RQ: research question
SUD: substance use disorder
TTM: transtheoretical model

## Data Availability

The datasets generated or analyzed during this study are not publicly available due to the potential sensitivity of the social media posts included in our study, to protect social media authors’ identities and anonymity.
